# Estrogen receptor beta as a prognostic factor in breast cancer patients: A systematic review and meta-analysis

**DOI:** 10.18632/oncotarget.7219

**Published:** 2016-02-06

**Authors:** Weige Tan, Qian Li, Kai Chen, Fengxi Su, Erwei Song, Chang Gong

**Affiliations:** ^1^ Guangdong Provincial Key Laboratory of Malignant Tumor Epigenetics and Gene Regulation, Sun Yat-Sen Memorial Hospital, Sun Yat-Sen University, Guangzhou, China; ^2^ Breast Tumor Center, Sun Yat-Sen Memorial Hospital, Sun Yat-Sen University, Guangzhou, China; ^3^ Collaborative Innovation Center for Cancer Medicine, Guangzhou, China

**Keywords:** estrogen receptor beta, breast cancer, survival, endocrine therapy, prognostic factor

## Abstract

**Background:**

The prognostic role of estrogen receptor beta (ERβ) in early-stage breast cancer is unclear. We performed a systematic review and meta-analysis to evaluate the prognostic value of ERβ in early-stage breast cancer patients.

**Method:**

We searched Medline, Embase, and the Web of Science for studies published between 1990 and 2015 that assessed ERβ status in breast cancer patients. A total of 25 studies comprising 9919 patients fitting our inclusion and exclusion criteria were included. The hazard ratios of ERβ status were extracted for diseases free survival (DFS)/) and overall survival (OS). Random or fixed-effects models were used when appropriate, and between-study heterogeneity was assessed.

**Results:**

In the 20 studies that assessed ERβ status using immunohistochemical (IHC) methods, we observed significantly improved DFS in patients positive for ERβ-1 (HR=0.56, 95%CI 0.40-0.78, *P*=0.0007) and ERβ-2 (HR=0.67, 95%CI 0.45-1.00, *P*=0.05). Improved OS was associated with a positive status for pan-ERβ (HR=0.60, 95%CI 0.45-0.80, *P*=0.0004) and ERβ-2 (HR=0.44, 95%CI 0.31-0.62, *P*<0.0001). In ERα-positive patients, ERβ positivity was not associated with DFS (HR=0.77, 95%CI 0.46-1.27, *P*=0.31) or OS (HR=0.64, 95%CI 0.37-1.11, *P*=0.11). In contrast, ERβ expression was significantly associated with increased DFS (HR=0.37, 95%CI 0.14-0.93, *P*=0.03) or OS (HR=0.44, 95%CI 0.30-0.65, *P*<0.0001) in ERα-negative patients. We did not observe an association between ERβ mRNA levels and DFS and OS.

**Conclusion:**

In this study, we showed that IHC ERβ status, rather than mRNA levels, is a prognostic factor that is associated with DFS and OS in breast cancer patients. The prognostic value of ERβ may be higher in ERα-negative patients than in ERα-positive patients.

## INTRODUCTION

Estrogen receptor α (ERα) has been established as a significant predictor of the response to endocrine therapy in breast cancer patients. Immunohistochemical (IHC) examination of ERα status is the standard-of-care pathological evaluation used to guide adjuvant endocrine therapy after surgery. Anti-estrogen approaches are recommended in ERα+ patients. The discovery of a second ER, ERβ, has lead to the re-evaluation of estrogen activity in normal mammary development, breast tumorigenesis and tumor progression. Despite over 15 years of research on ERβ, its clinical significance remains unclear. Mann et al. [1] were the first to report the significance of ERβ in predicting long-term clinical outcomes (e.g., disease-free survival) in breast cancer patients, a result confirmed by other studies [2-4]. However, conflicting findings suggest that ERβ status is not associated with survival [5, 6]. The aim of the present systematic review and meta-analysis was to investigate the association of ERβ status (positive vs. negative) and long-term clinical outcomes (e.g. disease-free survival, overall survival) of breast cancer patients.

## RESULTS

### Study characteristics

**Figure 1:**
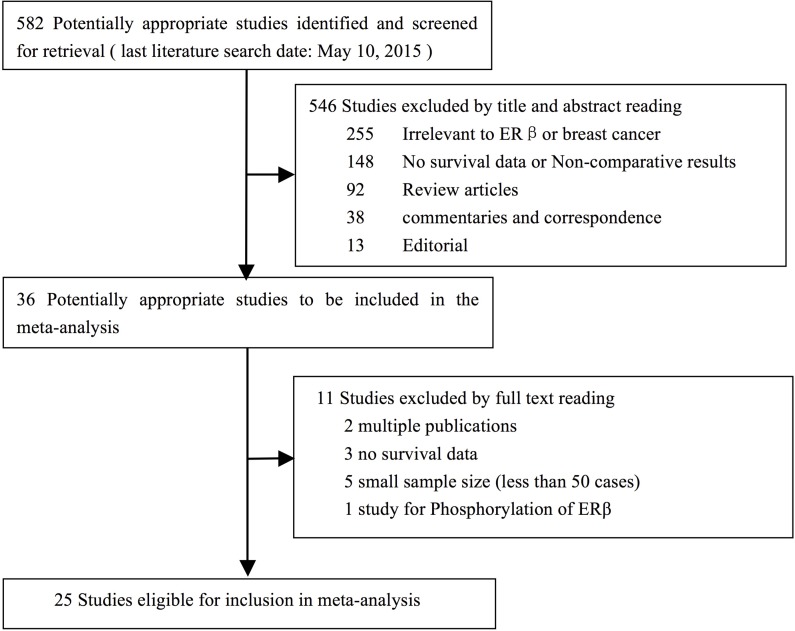
Flow diagram of studies identified, included, and excluded

Twenty-five studies [1-25] with the full text available were identified and included in this study (Figure 1). We examined the reference list of each study and did not identify any further studies for inclusion in our analysis. We included a total of 9919 patients from these studies. All publications were full-text articles. The features of the included studies are summarized in Table 1a, 1b. The mean patient age ranged from 48 to 68 years, and the median follow-up ranged from 27 to 174 months. Nine of the included studies had a quality score≥6. None of the included studies were prospective, randomized trials. All of the studies were retrospective and did not report any information about allocation concealment or blinding methods. The matching criteria varied among the studies. Most of the studies reported the length of the follow-up period, and 12 of them exhibited a sufficiently long follow-up (defined as a median follow-up time >60 months) for the outcomes to be determined. The treatment of missing data was not sufficiently described in most of the studies.

**Table 1a:** Features of included studies

References	Year	Patients (n)	Mean age	Methods •	ERb assessment ••	ERb status		Median Follow up(Months)	Quality Score
						ERb+	ERb−		
Borgquist et al.[11]	2008	512	64.2	i	ii	167	312	106	*****
		114 #				60	54	NA	
		139 ##				71	68	NA	
Chantzi et al.[20]	2013	95	52	i	i	b1:66 b2:65	b1:29 b2:30	NA	******
Gruvberger-Saal et al.[5]$, ¶¶¶¶	2007	425	NA	i	ii	262	91	174	*******
Guo et al. [21]¶¶¶¶¶	2014	490	49	i	ii	110	380	60	*******
Honma et al. [2]§	2008	442	56	i	ii	405	37	133	******
Hopp et al.[12]	2004	305	62	iii	v	141	164	65	*****
		186#				89	97	74	
		119 ##				52	67	50	
Kim et al.[13]	2012	139	NA	ii	iii	53	87	48	*****
Mahle et al.[14]§	2009	145	63	i	ii	129	16	165	*******
Mann et al.[1]	2001	47 ##	NA	i	ii	33	14	88	****
		118#	NA		ii	78	40	49	
Markey et al.[28]	2009	121	54	ii	iii	50	71	38	***
Myers et al.[15]	2004	150	NA	i	i	87	63	27	***
Nakopoulou et al.[3]	2004	181	61	i	ii	128	50	76	*****
Novelli et al.[6]	2008	936	NA	i	ii	520	416	50	*******
Omoto et al.[18]	2002	57	60.9	i	ii	15	42	48	***
Omoto et al.[17]	2001	88	54 &	i	i	52	36	NA	****
O'Neill et al. ¶[16]	2004	167	NA	i	ii	117	10	NA	******
			NA	ii	iii	86	35		
Palmieri et al.[19]	2004	82	59	i	i	33	46	96 ¶¶	****
Qui et al.[22]	2009	308	58	i	ii	123	185	48	***
Shaaban et al.[23]	2008	880	NA	i	i, ii	558	112	94	******
Sugiura et al.[24]	2007	150	53	i	i	103	47	58	***
				ii	iii	52	98		
Vinayagam et al. [4]¶,§	2007	141	68	i	i	100	41	BCS:71; BCR:79	*****
		100		ii	iii	34	30		
Wen et al.[25]	2002	116	53.7	iii	v	40	76	35.3	******
Wimberly et al.[26]	2014	Yale-1:649	NA	iv	iv	b1:228 b5:209	b1:228 b5:209	95	*****
		Yale-2:398				b1:147 b5:153	b1:148 b5:152	123	****
		Toronto: 976				b1:225 b5:153	b1:225 b5:153	98.2	****
		NCI-PBCS: 1375				b5:467	b5:468	116	****
Yan et al.¶¶¶,§[27]	2011	147	NA	i	ii	90	20	64	***
Zhang et al.[29]	2014	279	48.8	i	ii	40	109	92	***

• i.IHC; ii, PCR; iii, Immunoblot; iv. TMA

•• i, Allred score; ii, Proportion of positive cells; iii, Ct value; iv, AQUA score; v, Band intensities

# Tamoxifen/endocrine-treated subgroup

## untreated subgroup; & Median

¶ Postmenopausal patients.

¶¶ Estimated based on the description in the text.

¶¶¶ Familial breast cancer patients.

¶¶¶¶ Stage II patients.

¶¶¶¶¶ This group was reported in three publications involving the same study population. We selected the study with the longest follow-up period for analysis.

NA, Not available; ER, estrogen receptor;

**Table 1b:** Features of included studies

References	Year	Patients (n)	Antibody	ERa status		Tumor Burden		
				ERα+	ERα-	T1 %	N0 %	G3 %
Borgquist et al.[11]	2008	512	ERβ1: anti-mouse ERβ1 monoclonal antibody (EMR02; Novocastra)	407	72	63.1%	63.1%	NA
		114#		95	19	NA	NA	NA
		139##		114	25	NA	NA	NA
Chantzi et al.[20]	2013	95	ERβ1:anti-mouse ERβ1 monoclonal antibody (Clone PPG5/10; Serotec) ERβ2/cx: anti-human ERβ2 monoclonal antibody (Clone # 57/3; Serotec)	0	95	44.2%	56.8%	47.4%
Gruvberger-Saal et al.$, ¶¶¶¶[5]	2007	425	Pan-ERβ:anti-mouse ERβ monoclonal antibody (Clone 14C8; GeneTex) ERβ1:anti-mouse ERβ1 monoclonal antibody (Clone PPG5/10; Serotec)	248	105	26.6%	33.4%	NA
Guo et al. [2, 21]¶¶¶¶¶	2014	490	Pan-ERβ: Unclear (Fuzhou Maixin Biotechnology Development)	NA	NA	32.8%	51.2%	26.1%
Honma et al. §[2]	2008	442	Pan-ERβ:anti–rabbit polyclonal antibody (MYEB, M.Y) ERβ1:anti-mouse ERβ1 monoclonal antibody (Clone PPG5/10; DAKO) ERβ2/cx:anti-mouse ERβ2 monoclonal antibody (Clone # 57/3; Serotec)	364	78	39.4%	54.8%	NA
Hopp et al.[12]	2004	305	Pan-ERβ:anti-mouse ERβ monoclonal antibody (Clone 14C8; GeneTex)	272	33	23.9%	0.0%	43.7%
		186 #		176	10	26.9%	NA	40.7%
		119##		96	23	19.5%	NA	48.3%
Kim et al.[13]	2012	139	NA	139	0	61.4%	42.4%	20.7%
Mahle et al.§[14]	2009	145	Pan-ERβ:anti-mouse ERβ monoclonal antibody (Clone 14C8; GeneTex)	97	48	37.0%	51.7%	24.3%
Mann et al.[1]	2001	47##	Pan-ERβ:anti–rabbit polyclonal antibody (MYEB, M.Y)	30	17	NA	NA	NA
		118 #		75	43	NA	100.0%	NA
Markey et al.[28]	2009	121	NA	82	36	32.2%	45.5%	43.0%
Myers et al.[15]	2004	150	ERβ1:anti-mouse ERβ1 monoclonal antibody (Clone PPG5/10; Serotec)	123	27	NR	37.3%	49.3%
Nakopoulou et al.[3]	2004	181	ERβ1:anti-mouse ERβ1 monoclonal antibody (Clone PPG5/10; Serotec)	117	61	27.1%	38.1%	29.3%
Novelli et al.[6]	2008	936	Pan-ERβ:anti-mouse ERβ monoclonal antibody (Clone 14C8; Abcam) ERβ1:anti-mouse ERβ1 monoclonal antibody (Clone PPG5/10; GeneTex)	658	278	61.9%	57.6%	31.2%
Omoto et al.[18]	2002	57	Pan-ERβ: anti-rabbit ERβ polyclonal antibody βN; anti-chicken ERβ polyclonal antibody βT; ERβ1: anti-rabbit ERβ1 polyclonal antibody βC ERβ2/cx: anti-rabbit ERβcx polyclonal antibody	39	18	21.1%	62.5%	14.0%
Omoto et al.[17]	2001	88	ERβ1: anti-rabbit ERβ1 polyclonal antibody βC	62	26	22.7%	52.3%	4.5%
O'Neill et al.¶[16]	2004	167	ERβ1:anti-mouse ERβ1 monoclonal antibody (Clone PPG5/10; Serotec)	83	44	40.6%	53.3%	45.5%
			NA	79	42			
Palmieri et al.[19]	2004	82	Pan-ERβ:a purified polyclonal antibody ERβ2/cx: anti-ERβcx sheep polyclonal antibody	46	33	25.7%	53.2%	40.7%
Qui et al.[22]	2009	308	ERβ1:anti-rabbit ERβ polyclonal antibody(Ab-1, Oncogene research product)	198	110	42.2% &&	37.8%	39.6%
Shaaban et al.[23]	2008	880	ERβ1:anti-mouse ERβ1 monoclonal antibody (Clone PPG5/10; Serotec) ERβ2/cx: anti-human ERβ2 monoclonal antibody (Clone # 57/3; Serotec)	451	219	NA	NA	45.8%
Sugiura et al.[24]	2007	150	ERβ1: anti-rabbit ERβ1 polyclonal antibody ERβ2/cx: anti-rabbit ERβ2/cx polyclonal antibody	117	33	27.3%	60.4%	25.2%
			NA	117	33	27.3%	60.4%	25.2%
Vinayagam et al.¶,§[4]	2007	141	ERβ2/cx: anti-human ERβ2 monoclonal antibody (Clone # 57/3; Serotec)	98	43	44.7%	47.5%	43.3%
		100	NA	70	30	44.0%	49.0%	42.0%
Wen et al.[25]	2002	116	Pan-ERβ: anti-goat ERβ polyclonal antibody(Santa Cruz)	73	43	12.9%	37.1%	38.8%
Wimberly et al. [26]	2014	Yale-1:649	ERβ1: anti-mouse ERβ1 monoclonal antibody (PPG5/10; Thermoscientific) ERβ5: anti-human ERβ5 monoclonal antibody (Clone 5/25; Serotec)	246	208	28.0%	42.6%	NA
		Yale-2:398		158	102	54.8%	51.4%	
		Toronto: 976		288	118	65.3%	100.0%	
		NCI-PBCS: 1375		656	271	52.7%	59.0%	
Yan et al.¶¶¶,§[27]	2011	147	Pan-ERβ:anti-mouse ERβ monoclonal antibody (Clone 14C8; Abcam) ERβ1:anti-mouse ERβ1 monoclonal antibody (Clone PPG5/10; Genetex) ERβ2/cx: anti-human ERβ2 monoclonal antibody (Clone # 57/3; Serotec)	62	48	55.3%	69.9%	64.4%
Zhang et al.[29]	2014	279	ERβ1:anti-mouse ERβ1 monoclonal antibody (Clone PPG5/10; Serotec) ERβ2/cx: anti-human ERβ2 monoclonal antibody (Clone # 57/3; Serotec)	131	21	70.5%	41.6%	24.8%

# Tamoxifen/endocrine-treated subgroup;

## untreated subgroup; & Median;

$ Distant disease-free survival was considered to be disease-free survival in this study.

§ Breast cancer death and mortality were considered events affecting overall survival.

&& size<3 cm was considered T1-stage.

¶ Postmenopausal patients.

¶¶ Estimated based on the description in the text.

¶¶¶ Familial breast cancer patients.

¶¶¶¶ Stage II patients.

¶¶¶¶¶ This group was reported in three publications involving the same study population. We selected the study with the longest follow-up period for analysis.

NA, Not available; ER, estrogen receptor;

### The effect of ERβ on DFS

**Figure 2:**
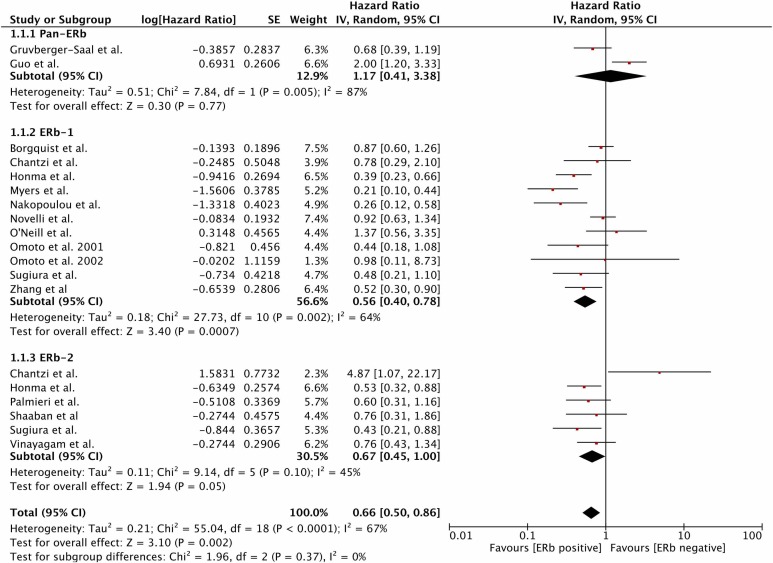
Prognostic role of IHC-determined ERβ status for DFS DFS, disease-free survival; IHC, immunohistochemistry; ER, estrogen receptor.

A total of 16 studies [2-7, 11-17, 19, 25] with available DFS used IHC as the method of ERβ assessment. Pooling the data showed that a positive status for ERβ-1(HR=0.56, 95%CI 0.40-0.78, *P*=0.0007; heterogeneity: *P*<0.01, I^2=64%) or ERβ-2 (HR=0.67, 95%CI 0.45-1.00, *P*=0.005; heterogeneity: *P*=0.10, I^2=45%) was significantly associated with improved DFS (Figure 2). Two studies [8, 21] used immunoblotting to assess pan-ERβ status. Pooling the data revealed that a positive pan-ERβ status was associated with an improved DFS (HR=0.51, 95%CI 0.35-0.75, *P*=0.0007; heterogeneity: *P*=0.33, I^2=9%; Figure S1). Five studies [4, 9, 12, 20, 24] assessed ERβ mRNA levels via PCR, and no association between total ERβ mRNA levels and DFS was detected (Figure S2). Wimberly et al. [22]employed a tissue microarray (TMA) to assess the pan-ERβ and ERβ-1 statuses of four independent populations. However, there was no association between ERβ status and DFS in these populations (Figure S3).

### The effect of ERβ on OS

**Figure 3:**
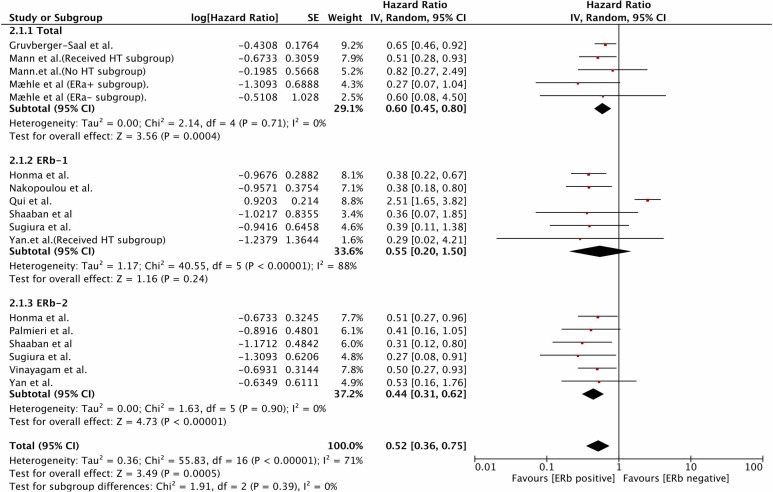
Prognostic role of IHC-determined ERβ status for OS OS, overall survival; IHC, immunohistochemistry; ER, estrogen receptor.

We pooled the data from 11 studies[1-5, 10, 15, 18-20, 23] with available overall survival data and observed that improved OS was associated with a positive status for pan-ERβ (HR=0.60, 95%CI 0.45-0.80, *P*=0.0004; heterogeneity: *P*=0.71, I^2=0%) and ERβ-2 (HR=0.44, 95%CI 0.31-0.62, *P*<0.0001; heterogeneity: *P*=0.90, I^2=0%), but not ERβ-1 (HR=0.55, 95%CI 0.20-1.50, *P*=0.24; heterogeneity: *P*<0.01, I^2=88%; Figure 3). After excluding the study reported by Qui et al. [18], a positive ERβ-1 status was shown to be associated with improved OS without significant heterogeneity(HR=0.38, 95%CI 0.25-0.57, heterogeneity: *P*=1.00, I^2=0%). When the data from the two studies [8, 21] that used immunoblotting to assess pan-ERβ status were pooled, we observed an association between a positive pan-ERβ status and improved OS (HR=0.62, 95%CI 0.46-0.84, *P*=0.002; heterogeneity: *P*=0.11, I^2=55%; Figure S4). There were 3 studies [4, 20, 24] that assessed the mRNA levels of ERβ using PCR; we found no association between total ERβ mRNA levels and OS (Figure S5).

### ERα as an effect modifier

**Figure 4:**
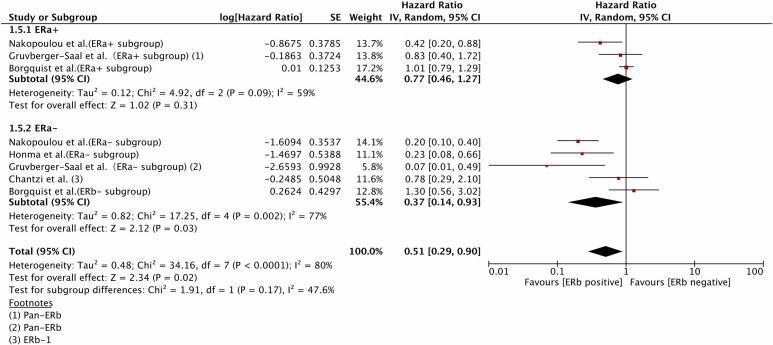
The prognostic role of IHC-determined ERβ status for DFS varied by ERα status DFS, disease-free survival; IHC, immunohistochemistry; ER, estrogen receptor.

**Figure 5:**
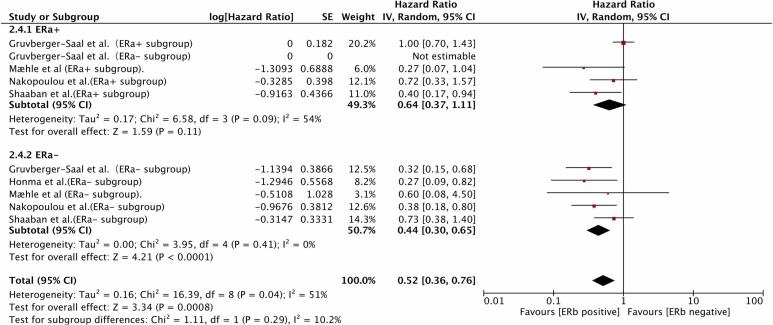
The prognostic role of IHC-determined ERβ status for OS varied by ERα status OS, overall survival; IHC, immunohistochemistry; ER, estrogen receptor.

A total of 7 studies [2, 3, 5, 7, 10, 19, 25] reported the HR of the IHC-determined ERβ status (pan-ERβ/ERβ-1/ERβ-2) for DFS and OS in ERα-positive or negative patient subgroups. In ERα (+) patients, ERβ status was not associated with DFS (HR=0.77, 95%CI 0.46-1.27, *P*=0.31; heterogeneity: *P*=0.09, I^2=59%) or OS(HR=0.64, 95%CI 0.37-1.11, *P*=0.11; heterogeneity: *P*=0.09, I^2=54%). In fact, Zhang [25] found that a positive ERβ status was correlated with improved DFS in univariate, but not multivariate analysis. Vinayagam [4] reported that ERβ status was not correlated with DFS, but the associated HR was not available, and this study was therefore not included in the afore mentioned meta-analysis. In contrast, a positive ERβ status was significantly associated with increased DFS (HR=0.37, 95%CI 0.14-0.93, *P*=0.03; heterogeneity: *P*<0.01, I^2=77%) and OS (HR=0.44, 95%CI 0.30-0.65, *P*<0.0001; heterogeneity: *P*=0.41, I^2=0%) in ERα (−) patients (Figures 4 & 5).

### Sensitivity analysis and publication bias

A sensitivity analysis revealed that a positive ERβ (pan ERβ/ERβ-1/ERβ-2) status was significantly associated with improved DFS or OS in studies with a median follow-up time greater than 60 months [1-8, 10, 15, 17, 19, 20, 22, 23] (Table S1). ERβ-1 was not associated with DFS or OS in studies with a sample size ≥ 200 [2, 5-8, 17-19, 25]. The funnel plots for the studies for DFS were symmetric, indicating no publication bias (Figure S6). However, the distribution of the OS funnel plots was not symmetric. As shown in Figure S7, the studies focusing on specific ERβ (ERβ1/ERβ2) and pan-ERβ reactivity were mostly located on the left and right sides of the funnel plot, respectively, indicating possible publication biases.

## DISCUSSION

ERβ was discovered nearly two decades ago, but its role as a prognostic or predictive factor in breast cancer remains elusive. Most studies examining ERβ as a biomarker have been retrospective, and these studies have used a variety of detection methods, leading to discrepant results. IHC is the most common method employed for ERβ assessment. In this meta-analysis study, we observed that a positive ERβ status, as assessed via IHC, was generally associated with improved DFS and OS. Multiple ERβ isoforms (ERβ-1, ERβ-2/cx) arise via alternative splicing of downstream coding exons or posttranslational proteolysis [26, 27]. In this study, we noted that ERβ-2 was associated with improved DFS and OS. In contrast, ERβ-1 was associated with DFS, but not OS, which may be attributed to a study by Qui et al. [18], who provided the only report of an association between positive ERβ-1 status and a poorer OS. After the exclusion of this study, the pooled HR(95%CI) of ERβ-1 for OS changed significantly, from 0.55(95%CI: 0.20-1.50) to 0.38(95%CI: 0.25-0.57). The heterogeneity of the data synthesis was also eliminated. After careful examination, we noted in consistent results within Qui et al.'s study. In their report, they indicated that ERβ-positive patients exhibit a significantly worse overall survival prognosis compared with ERβ-negative patients. However, when stratified by HER2 status, the survival curves of the ERβ-positive and ERβ-negative patients overlapped in both strata. The authors did not attempt to explain this result. We therefore suggest that the exclusion of this study from our meta-data analysis is appropriate.

### Assessment method and clinical outcomes

Various methods had been used to assess ERβ status. Two studies employed immunoblotting as the detection method and revealed that a positive ERβ status was associated with improved DFS, similar to studies employing IHC [8, 21]. However, a study by Wimberly et al. [22]showed no association between ERβ status and DFS when TMA was used to assess ERβ status in four large-cohort populations. We speculate that TMA may not be an accurate method for ERβ assessment. Its major limitation is that the small cores employed to construct a TMA may not accurately and comprehensively represent the whole tissue specimen. Eckel-Passow et al. [28]reported that the number of TMA cores necessary to adequately represent the whole tissue specimen is biomarker-specific. They showed that 2-3 cores appeared to be adequate for assessing the status of B7-H3, Ki-67, CAIX, and IMP3 expression in renal cancer patients, whereas as many as 10 cores were insufficient for assessing B7-H1. Thus, the association between B7-H1determined in whole tissue sections and renal cancer-specific death is not easily revealed through TMA assessment.

Several studies found no consistent association between the mRNA and protein levels of ERβ [14, 29, 30]. Furthermore, an inverse association between ERβ mRNA levels and improved survival has been reported. Speirs et al. [31]noted that ERβ mRNA levels were increased in tamoxifen-resistant breast cancer patients. Similarly, Kim et al. [9] reported that a higher ERβ mRNA level is associated with poorer DFS in patients treated using endocrine therapy. We believe that the assessment of ERβ status based on mRNA levels may be inaccurate because samples from breast tissue might contain cells from surrounding cancerous tissue. Furthermore, post-transcriptional regulation may also compromise the prognostic value of ERβ mRNA [32]. In our meta-analysis, we found no association between ERβ mRNA levels and survival (DFS or OS). Hence, ERβ mRNA status does not appear to be promising for clinical use.

### Prognostic role of ERβ varied by ERα status

As noted above, the prognostic value of ERβ varies depending on a patient's ERα status. The mechanism underlying this effect may be the molecular interplay between ERα and ERβ. Charn et al. [33] investigated the effects of ligand-occupied and unoccupied ERα and ERβ on chromatin binding. They showed that although ERα and ERβ restrict each other's binding site occupancy, ERα is dominant. The binding sites of ERα and ERβ overlap substantially when the are present alone. However, when both ERα and ERβ are present, only a few binding sites are shared. When both receptors are present, ERα displaces ERβ and shifts ligand binding to sites that are less enriched in the estrogen response element. This finding supports our observation that in ERα+ patients, the prognostic role of ERβ was less significant than in ERα- patients. Because endocrine therapy is administered to ERα+, but not ERα-, patients, we suggest that endocrine therapy may play a role as an effect modifier. Unfortunately, there are insufficient data to perform a meta-analysis addressing this issue. Novelliet al.[6]reported that in patients who receive endocrine therapy, a positive ERβ status is associated with increased DFS. Similar results have been reported by other investigators [1, 8]. However, Yan et al. [23] found that a positive ERβ status was associated with improved OS in univariate, but not multivariate, analyses. O'Neil et al. [12]noted a trend (though not statistically significant)toward poorer DFS in patients with a positive ERβ status. Hence, the predictive role of ERβ for the endocrine therapy response is unclear, due to the conflicting results provided by different studies [1, 6, 7, 12, 23].

No association between ERβ status and DFS/OS was observed in patients who did not receive endocrine therapy [1, 7, 23]. We believe that the sample sizes of these studies are too small to detect an association. Our group has initiated a multicenter randomized double-blind prospective clinical trial comparing the efficacy of tamoxifen as an adjuvant endocrine therapy in early-stage ERα/PR-/ERβ+ breast cancer patients (ClinicalTrials.gov Identifier:NCT02062489). Sun et al. has initiated a similar multicenter study, in which early stage, triple-negative breast cancer patients are randomized into a toremifene/anastrozole group or an observation group (ClinicalTrials.gov Identifier: NCT02089854)

### DFS and OS as clinical endpoints

We observed heterogeneity in the synthesis of the HR of pan-ERβ or ERβ-1 status for DFS. However, there was no heterogeneity in the synthesis of the HR of pan-ERβ, ERβ-1 (with the exception of Qui's study) or ERβ-2 status for OS. We suggest that this discrepancy may be due to the definition of DFS/OS. OS is a universally accepted measure of the clinical benefit of a treatment and can be precisely measured. As a result, there might be less heterogeneity for OS. In contrast, the definition of DFS varies between studies. For example, in the NSABP B-06 study [34], DFS was defined as the first recurrence of disease at a local, regional, or distant site, and the diagnosis of a second cancer and death without evidence of cancer were considered DFS events. In contrast, the guidelines from the DATECAN initiative (Definition for the Assessment of Time-to-event Endpoints in CANcer trials) [35] recommend that DFS should include death of from breast cancer as an event. Most of our included studies did not specify the definition of DFS, which may have resulted in heterogeneity in the synthesis of HR of DFS.

### Publication bias

All of the included studies were retrospective and may be subject to publication bias. Insignificant HRs, especially following multivariate analysis, are less likely to be reported in retrospective studies. In the present study, we obtained asymmetric funnel plots for the synthesis of the HRs for OS. Studies reported significant HRs of ERβ-1 and ERβ-2 for OS tend to fall on the left side of the reference line, indicating that insignificant HRs are less likely to be reported. Several studies [4, 6, 23, 36] reported finding no association between ERβ status and OS, but without an available HR and/or 95%CI. Hence, we must be cautious about the prognostic role of ERβ for overall survival.

### Limitations

Several additional limitations should be addressed. First, IHC was commonly used for detecting ERβ status in most studies, but different hospital used varied commercial antibodies and didn't have uniform criteria. Reported data show that many commercially available IHC stains for ERβ have cross-reactivity with ERα [37]. Percentage of immunoreactive cells and allred scores were used to assess ERβ status, while the cut-off values varied from 1% to 25% (Percentage of immunoreactive cells) [6, 14, 17], and from 2-4 (Allred score) [2, 11, 15] across different studies. Different cut-off values used by different studies may cause limitation to our analysis. Second, some of the HRs were not available from the full-text of the included study, and were extrapolated from survival curves. Although, this method has been demonstrated to be feasible [38, 39], we still consider this as a limitation. Additionally, HRs for synthesis in our analysis were derived from univariate and/or multivariate analysis (Table S2). This is also a major limitation, as the most standard approach should be collecting HRs derived from prospective controlled trials, with multivariate analysis adjustment.

## CONCLUSION

In this meta-analysis, we showed that ERβ status, determined via IHC,is generally associated with DFS/OS in breast cancer patients. Assessment of ERβ mRNA levels is not recommended. As a prognostic factor, ERβ may be more important in ERα (+) patients than ERα (−) patients. Based on these findings, we recommend the initiation of a prospective study to confirm the prognostic value of ERβ in breast cancer patients.

## MATERIALS AND METHODS

This study was waived the full IRB review of Sun Yat-sen Memorial Hospital, based on the institutional policy. This study was also performed according to the recommendations of the Cochrane Collaboration and the Quality of Reporting of Meta-analysis guidelines (MOOSE) and reported according to the PRISMA statements [40, 41].

### Study selection

We searched Medline, Embase, and the Web of Science for potentially relevant studies. The following keywords were searched in the “Title” or “Abstract”: “Estrogen receptor,” “Beta,” and “Breast cancer,” without restrictions on the region and publication type. English language was requied for publication. We manually searched the retrieved articles to identify relevant studies. When multiple publications reported on the same study population, the report that was most complete or that had the longest follow-up period was used. The last date of the search was May 10^th^, 2015.

### Inclusion and exclusion criteria

Studies were eligible if they met the following criteria: (1) the main exposure of interest was early-stage breast cancer stratified by ERβ status (negative/positive or low/high expression); (2) the outcome of interest was disease-free survival or overall survival; (3) hazard ratios (HRs) with corresponding 95% confidence intervals (CIs) or survival curves for ERβ were reported; and (4) over 50 patients were enrolled in the study, which did not present redundant data.

### Data extraction

Two reviewers (C.G. & W.T.) independently extracted the data from the included studies. Any disagreement was resolved by the third author (E.S.). The following data were collected: first author, year of publication, clinicopathological features of the study population, methods of ERβ assessment, number of included patients, and the reported outcomes. The outcomes assessed included disease-free survival (DFS) and overall survival (OS) in patients with different ERβ statuses. We assessed the quality of the included studies using the Newcastle-Ottawa quality assessment tool [42]. We allocated a score of 0-9 to each included study, and those with a score≥6 were considered to be of high quality.

### Statistical analysis

The hazard ratio (HR) was used as a summary statistic for survival analysis, as described by Parmar and colleagues [43]. An HR of less than 1 indicated a survival benefit favoring ERβ+ patients. We used a random-effects model for this meta-analysis. The data were pooled and weighted using generic inverse variance. Heterogeneity between studies was assessed with the χ2 and I2 statistics. When higher values of the χ2 and I2 statistics (>50%) indicated heterogeneity between studies, we applied sensitivity and subgroup analyses to further evaluate the heterogeneity. We performed a sensitivity analysis when the outcome of interest was reported in more than 3 studies. We used funnel plot analyses to analysis to determine publication bias. A two-tailed p value of less than 0.05 was considered statistically significant. Statistical analyses were performed with Review Manager Version 5.3

## SUPPLEMENTARY MATERIAL FIGURES AND TABLES


