# Supplementary material for: Estrogen receptor beta as a prognostic factor in breast cancer patients: A systematic review and meta-analysis
Source: Oncotarget. 2016 Feb 6;7(9):10373–85. doi: 10.18632/oncotarget.7219 (PMC4891126; doi:10.18632/oncotarget.7219)
Supplement: Supplementary file 1 [file oncotarget-07-10373-s001.pdf]

Estrogen receptor beta as a prognostic factor in breast cancer patients: A systematic review and meta-analysis

Supplementary Material

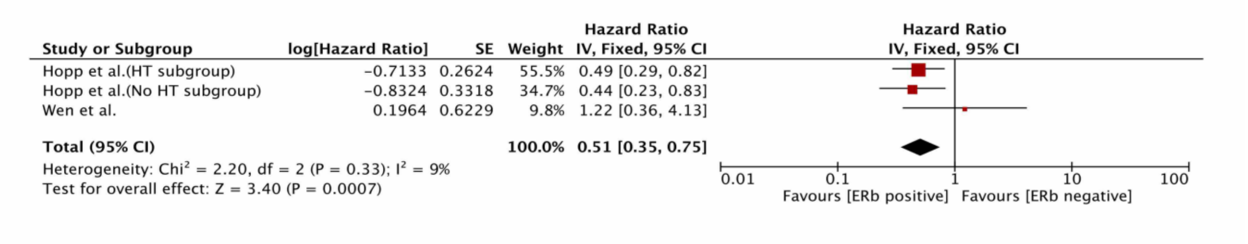

Figure S1. Prognostic role of IB-determined ERβ status for DFS. DFS, disease-free survival; IB, immunoblot; ER, estrogen receptor.

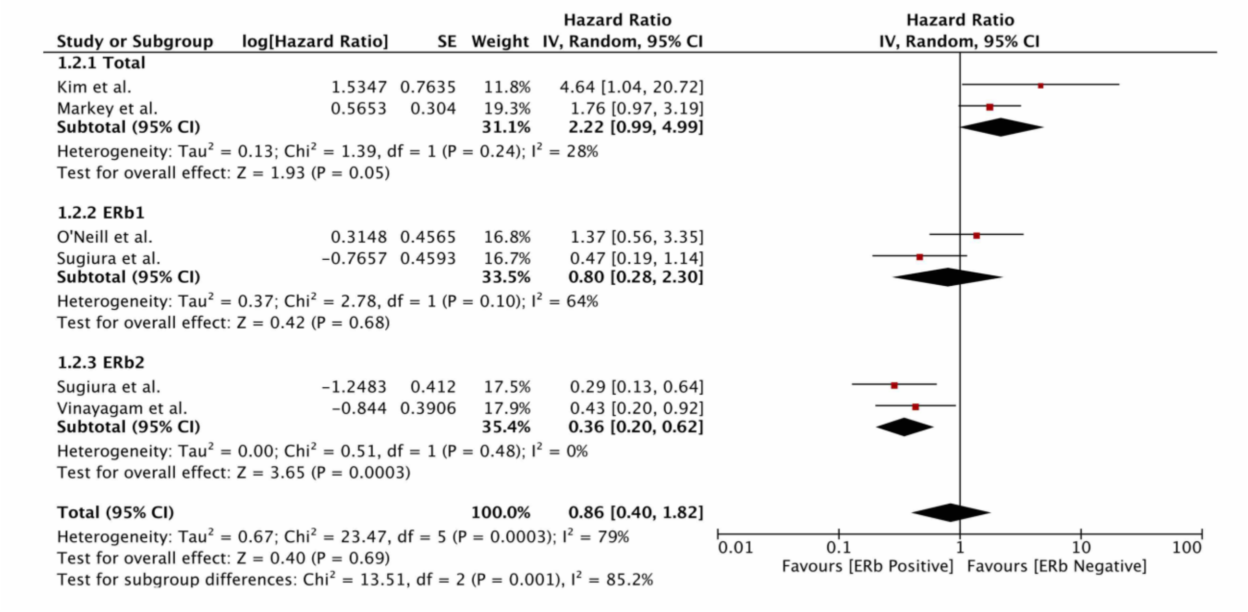

Figure S2. Prognostic role of PCR-determined ERβ status for DFS. DFS, disease-free survival; PCR, polymerase chain reaction; ER, estrogen receptor.

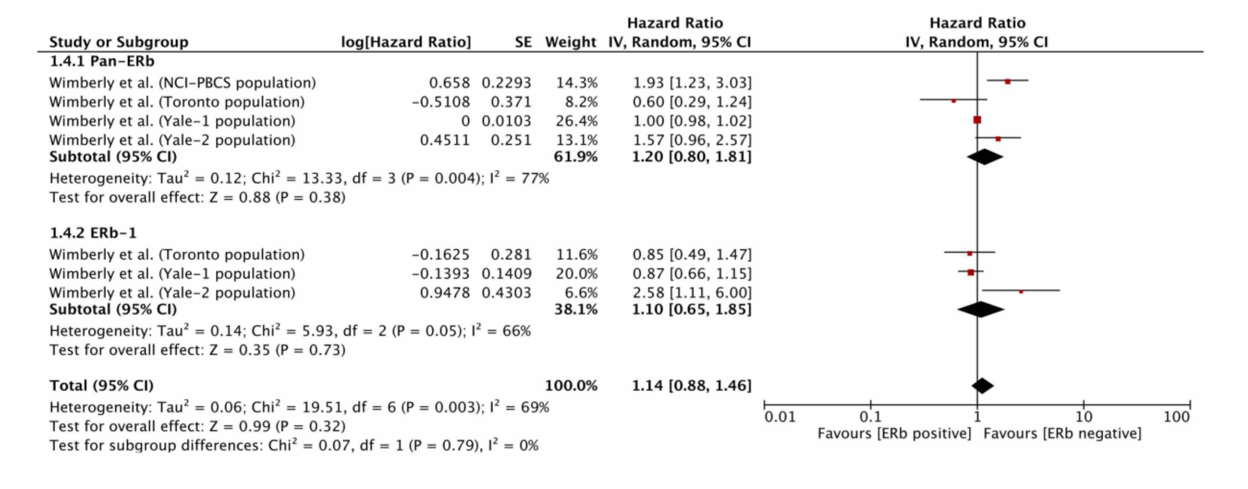

Figure S3. Prognostic role of TMA-determined ERβ status for DFS. DFS, disease-free survival; TMA, tissue microarray; ER, estrogen receptor.

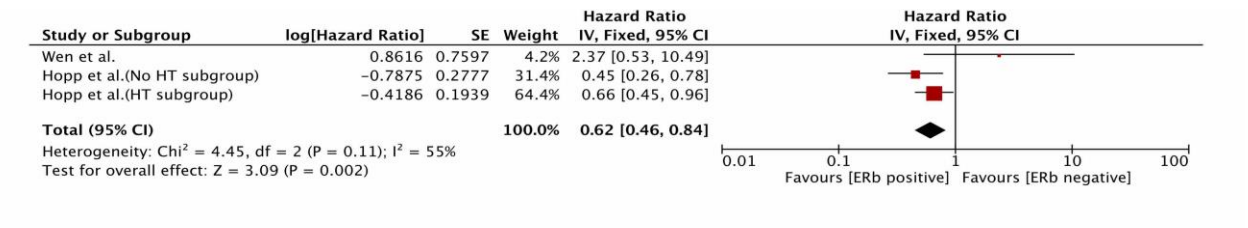

Figure S4. Prognostic role of IB-determined ERβ status for OS. OS, overall survival; IB, immunoblot; ER, estrogen receptor.

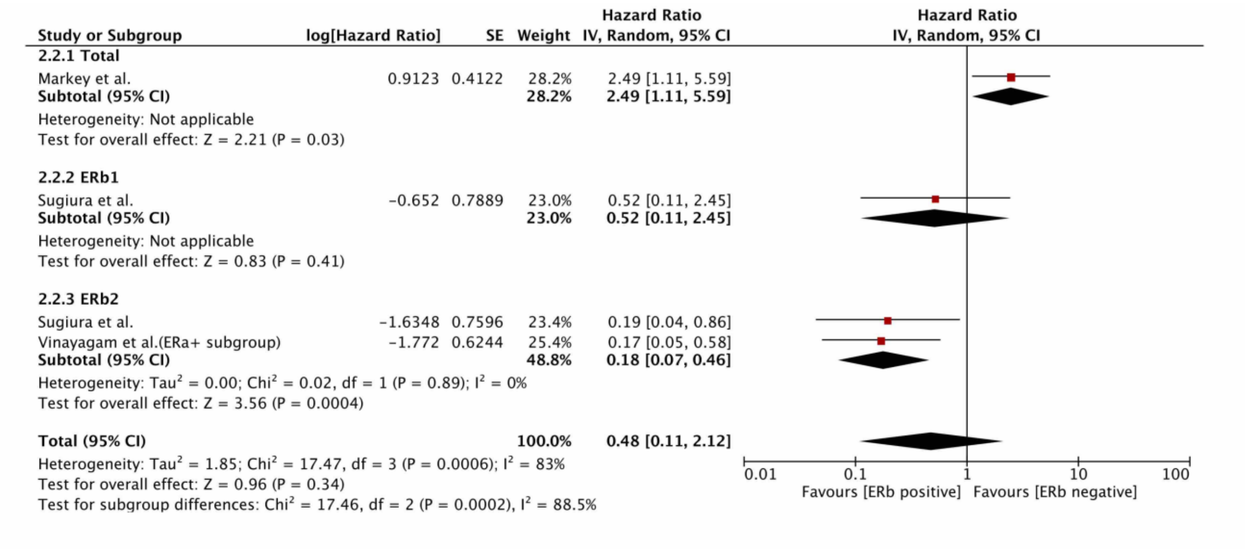

Figure S5. Prognostic role of PCR-determined ERβ status for OS. OS, overall survival; PCR, polymerase chain reaction; ER, estrogen receptor.

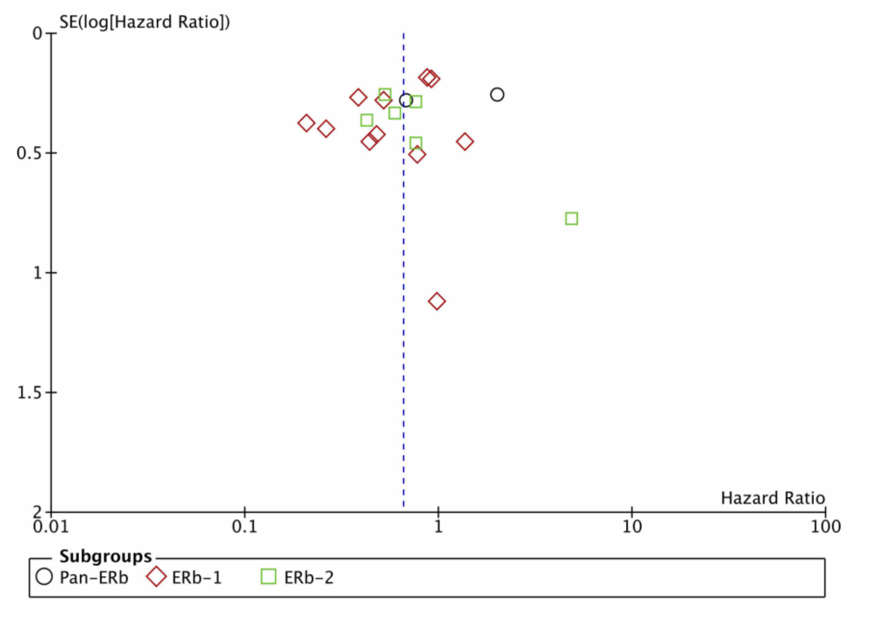

Figure S6. Funnel plots for assessing publication bias for DFS. Only studies assessing ERβ status using IHC were included. DFS, disease-free survival; IHC, immunohistochemistry.

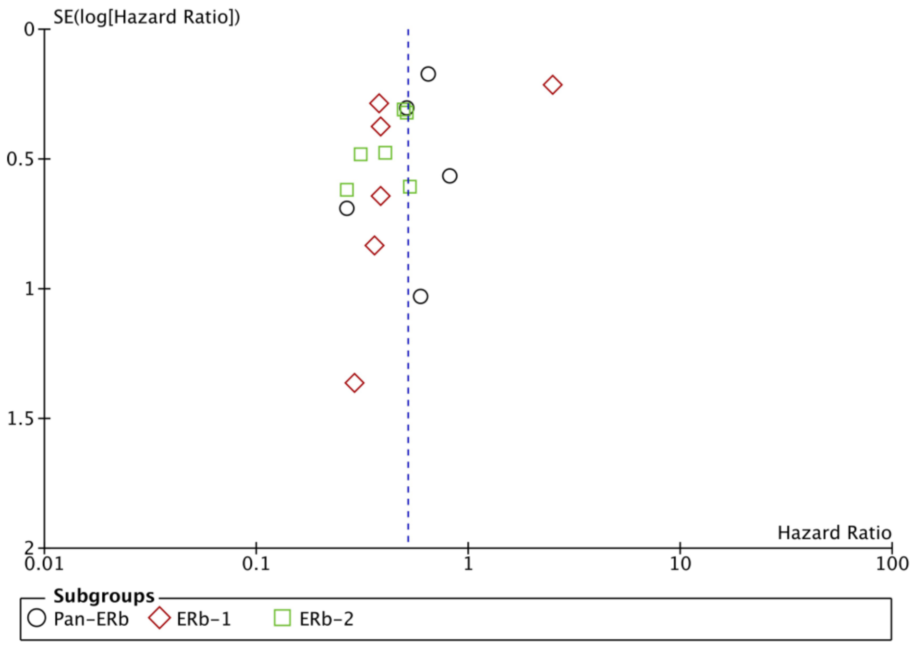

Figure S7. Funnel plots for assessing publication bias for OS. Only studies assessing ERβ status using IHC were included. OS, overall survival; IHC, immunohistochemistry.

Supplementary Table S1: Sensitivity analysis of survival in patients with different ERb(Total/ERb1/ERb2) status (IHC).

Endpoints	Pan-ERb				ERb-1				ERb-2			
	Number of studies	Random effect	Study heterogeneity		Number of studies	Random effect	Study heterogeneity		Number of studies	Random effect	Study heterogeneity	
		HR(95%CI)	I^2	p		HR(95%CI)	I^2	p		HR(95%CI)	I^2	p
DFS												
Median follow-up >60 months	N/A				4	0.49(0.30,0.82)	72 %	0.010	4	0.63(0.46,0.86)	0%	0.79
Sample size≥200	N/A				4	0.72(0.48,1.07)	61 %	0.050	N/A			
OS												
Median follow-up >60 months	3	0.60(0.45,0.80)	0%	0.71	4	0.38(0.25,0.58)	0%	1.000	5	0.46(0.32,0.65)	0%	0.92
Sample size≥200	N/A				3	0.76(0.16,3.47)	93 %	<0.001	N/A			

NA, not available.

Supplementary Table S2: Origination and classification of HRs for DFS and OS.

References	Year	Data for DFS	Data for OS
Vinayagam et al.	2007	i,ii	i ,i*,ii
Honma et al.	2008	i ,i*,ii	i ,i*,ii
Novelli et al.	2008	i ,i*	
Gruvberger-Saal et al.	2007	i*,ii	i*
Mann et al.	2001		i
Mahle et al.	2009		i*,ii
Hopp et al.	2004	i,ii	i*,ii
Borgquist et al.	2008	i ,i*	
Palmieri et al.	2004	i,ii	i,ii
Shaaban et al.	2008	ii	i*,ii
Nakopoulou et al.	2004	i*,ii	i*,ii
Myers et al.	2004	i*	
Sugiura et al.	2007	i*,ii	i*,ii
Omoto et al.	2002	i*	
Omoto et al.	2001	i*	
Yan et al.	2011		i*
Zhang et al.	2014	i*	
O'Neill et al.	2004	i*	
Guo et al.	2014	i*,ii	
Chantzi et al.	2013	i,ii	
Qui et al.	2009	i*	i*
Markey et al.	2009	i*	i*,ii
Kim et al.	2012	i*,ii	
Wen et al.	2002	ii	ii
Wimberly et al.	2014	i*,ii	

i Original data dervived from univariable analysis ;

i* Extracted data from suvival curves as univariable analysis;

ii Original data dervived from multivariable analysis ;
